# Cognitive reserve and mild cognitive impairment in older adults of low socioeconomic status: evidence from an observational study in Colombia

**DOI:** 10.1093/geronb/gbaf131

**Published:** 2025-07-08

**Authors:** Juan Felipe Martínez Flórez, Lina Marcela Bernal Sandoval, Oscar Armando Erazo Santander, César Mejía Zuluaga

**Affiliations:** Faculty of Health, Universidad Santiago de Cali, Cali, Colombia; Faculty of Health, Universidad Santiago de Cali, Cali, Colombia; Department of Psychology, School of Social Sciences & Humanities, Universidad Pontificia Bolivariana Sede Monteria, Monteria, Colombia; Psychology Laboratory, Faculty of Humanities & Social Sciences, Universidad de San Buenaventura, Cali, Colombia

**Keywords:** Cognitive resilience, Health disparities, Neuropsychological assessment, Socioeconomic determinants of health

## Abstract

**Objectives:**

The construct of cognitive reserve (CR) suggests that environmental factors influence cognition over time, resulting in a more resilient response to pathology or adverse conditions in some individuals. The goal of this study was to identify and analyze differences in CR among older adults of low socioeconomic status (SES).

**Methods:**

A sample of 102 older adults, both with (*n* = 52) and without (*n* = 50) amnestic mild cognitive impairment (aMCI), underwent a cognitive assessment protocol including the Cognitive Reserve Index Questionnaire (CRIq). Participants’ SES levels were classified using the European Society for Opinion and Marketing Research Standard Demographic Classification. Mean and distributional comparisons, logistic regression, and receiver operating characteristic (ROC) analysis were conducted.

**Results:**

Mean comparisons and distribution analysis showed that participants with aMCI had lower CR than those without aMCI. Logistic regression models revealed that CRIq score predicted aMCI in this population (OR = 0.955, *p* < .001), particularly through education (OR = 0.546, *p* < .001) and work (OR = 0.970, *p* < .001) dimensions. ROC curve results indicate the model has adequate discriminatory power, with an area under the curve (AUC) of 0.738.

**Discussion:**

Low CR is a sign of pathological cognition in low SES older adults. A higher level of CR in subjects with low SES, even if not meeting the criteria for High CR, has a role in mitigating aMCI. Future studies expand on these findings by examining the relationship between CR and SES in the brain-behavior association, including biomarkers such as the A/T/N framework.

Human ageing involves a combination of physiological changes that lead to a decline in physical, social, and cognitive function, increasing the risk of disease. Within cognitive aging, amnestic mild cognitive impairment (aMCI) is often considered an intermediate stage between normal ageing and Alzheimer’s disease (AD) (Petersen, 2016). This condition, characterized by memory loss, is influenced by physical and environmental factors and can show unique variations between individuals. Older adults with aMCI experience more pronounced memory loss or thinking problems than are typical for their age, but retain overall cognitive function and normal performance in daily activities. Proposed diagnostic criteria for aMCI include subjective complaints of memory changes, confirmed by a reliable informant, and objective evidence of memory impairment, demonstrated by performance below normative values for age and education (Petersen et al., 2018). Age-related cognitive and memory impairment has been explained by changes in multiple brain structures and their connectivity, such as the hippocampus, frontal cortex, thalamus, prestriatal circuitry, and temporal and parietal lobe areas (Cai et al., 2017; Dahan et al., 2020).

The construct of “cognitive reserve” (CR) has gained prominence in recent years in research on aging and cognition. This construct aims to explain discrepancies between clinical outcomes and observed brain decline by proposing its understanding through the relationship between risk and protective factors in lifestyle. In this sense, CR suggests that environmental factors influence cognition over time, potentially predicting and explaining why some individuals show a more resilient response to pathology or adverse conditions (Baltes & Kuhl, 1992; Stern, 2021).

Due to the wide range of variables that can affect cognition, there is currently extensive debate about a unified definition of CR and the correct way to estimate it (Kartschmit et al., 2019). However, there is relative consensus on the assumption of CR as “adaptability that helps to explain the differential susceptibility of cognitive abilities or daily functioning to brain ageing, pathology, or insult” (Stern et al., 2020, p. 5). This definition assumes that CR has correlates in brain structure that are integrated by flexibility, compensation, and optimization of the functioning of different neural networks. Studies using Magnetic Resonance Imaging (MRI) have identified structural brain changes in healthy individuals with high levels of CR. These individuals exhibit increased cortical grey matter volume density in regions such as the mid-frontal gyrus, angular gyrus, anterior cingulate of the frontal lobe, right superior temporal gyrus, and left insular cortex of the temporal lobe (Arenaza-Urquijo et al., 2013; Conti et al., 2021; Stern, 2017). By contrast, individuals with mild cognitive impairment (MCI) have shown reduced grey matter density in areas such as the parahippocampal gyrus and the right middle cingulate gyrus (Zhu et al., 2021; Zhou et al., 2024). Furthermore, functional connectivity analyses using functional MRI have also demonstrated positive associations between high CR and stronger functional connectivity in regions associated with the default mode network and the dorsal attention network in healthy individuals (Anthony & Lin, 2018; Franzmeier et al., 2018). These neural correlates depend both on the individual’s environmental context and age, and on the ability of the nervous system to respond to intrinsic or environmental stimuli by reorganizing its structure, function, and connections. Cognitive reserve aims to understand brain function from a dynamic or efficient model, in which alternative or complementary brain networks are used to counteract damage in specific areas, facilitating the effective performance of tasks or the maintenance of a clinical state considered normal, through the process called compensation.

Recent contributions within the domain of neuroscience have emphasized the influence of sociodemographic and socioeconomic factors on the brain health of older adults (Marden et al., 2017). Research indicates that older adults with low socioeconomic status (SES) are more susceptible to developing mild cognitive impairment (MCI) and other cognitive dysfunctions (Hernandez et al., 2024). This disparity in cognitive health is more pronounced in low- and middle-income regions, such as Latin America, where a significant correlation between sociodemographic factors and the risk of cognitive impairment in old age has been documented (Ibanez et al., 2021; Sarmiento Buitrago et al., 2024). Socioeconomic factors that affect cognitive functioning in ageing include educational level, place of residence (urban or rural), economic disparity, and sedentary lifestyle (Baez et al., 2023; McGlinchey et al., 2024). A recent study examined the impact of SES on the rate of functional decline over 8 years across six domains, involving 5,018 individuals with a mean baseline age of 64.44 years. The results indicate that lower SES is associated with accelerated aging in various functional abilities and phenotypes, independent of the presence of health problems (Darin-Mattsson et al., 2017).

The concept of CR, which pertains to the potential for enhanced cognitive functioning in aging, is frequently examined in the context of educational attainment, healthy living habits, access to resources, and lifestyles. However, it should be noted that the global average for years of education is only 8.7, indicating that a significant portion of the world’s population is not highly educated (UNDP [United Nations Development Programme], 2024). Furthermore, the World Bank (2024) reports that low and middle-income countries are home to 75% of the world’s population and 62% of the world’s poor. In this context, it is pertinent to inquire whether this substantial segment of the population with limited educational attainment and SES falls outside the purview of the benefits typically associated with CR.

The present study focuses on the study of cognitive impairment in ageing subjects of low SES and seeks to understand the role of CR in this population. Specifically, the objective is to ascertain whether there are disparities in CR among low SES older adults with and without aMCI. Drawing upon extant literature concerning SES and CR, it is hypothesized that CR may exert a protective effect on cognition in older adults of low SES.

## Method

### Participants and procedure

A total of 102 participants aged 65 years and over were recruited from the outpatient and resident services of the Geriatric Hospital “San Miguel” in the city of Cali, Colombia, to take part in this study. The inclusion criteria for both groups were as follows: subjects had to be 65 years of age or older, not report neurological or psychiatric disease, not report a history of psychoactive substance use, and be healthy, as reported in a semi-structured clinical interview and self-reported questionnaire. The study was approved by the Ethics Committee of the Faculty of Health of the Universidad Santiago de Cali and the Geriatric Hospital “San Miguel” of Cali, Colombia. The study was conducted in accordance with the ethical principles of the Declaration of Helsinki (2013). All participants provided written informed consent to participate in the study.

The Standard Demographic Classification of the European Society for Opinion and Marketing Research (ESOMAR) was utilized to categorize the SES of the sample (Wolf & Hoffmeyer-Zlotnik, 2003). This questionnaire has consistently demonstrated its effectiveness in evaluating SES across diverse international populations, including Latin American studies (Celis-Morales et al., 2012; Martínez-Flórez et al., 2024; Migeot et al., 2022). The estimation of SES by ESOMAR is derived from a dimensional framework incorporating data on the educational attainment and occupation of the primary household income earner. For non-working participants, economic status is assessed based on specific material possessions. European Society for Opinion and Marketing Research’s categorization of SES encompasses six levels: A (very high) to E (low). The present study included participants classified as D (lower-­middle) or E (low). In accordance with the findings of earlier studies (Martínez-Flórez et al., 2024; Migeot et al., 2022), a consensus was reached to consolidate levels D and E into a composite low SES category. To focus on lower SES participants, we excluded participants categorized as A, B, Ca, or Cb.

To identify participants with aMCI at the time of recruitment, we applied the clinical core criteria proposed by the NIA-AA guidelines. (Albert et al., 2011; Jack et al., 2018; Petersen et al., 2013). The aforementioned criteria encompassed the following: the presence of a subjective complaint regarding memory, accompanied by concern about memory or general cognitive decline in the past year relative to a preexisting pattern; the presence of objective cognitive impairment in one or more cognitive domains (−1.5 SD compared to normative data); the preservation of functional independence in daily activities; and the absence of signs of dementia. The general cognitive function of the subjects was assessed using the Addenbrooke’s Cognitive Examination Revised (ACE-R) (Mioshi et al., 2006). The presence of objective memory impairment was determined by examining the delayed recall scores from the Rey Auditory Verbal Learning Test (RAVLT) (Bean, 2011) and the Memory domain of the ACE-R. The Instrumental Activities of Daily Living Scale (IADL) (Lawton & Brody, 1969) and the Physical Self-Maintenance Scale (PSMS) (Pfeffer et al., 1982) were used to confirm preserved functional abilities. The Geriatric Depression Scale (GDS) (Yesavage et al., 1982) was administered to screen for depression, and the Hachinski Ischemic Score (HIS) (Pantoni & Inzitari, 1993) to rule out signs of vascular pathology. It is important to note that all the aforementioned tests had previously been translated and adapted to the Colombian or Latin American population. Cutoff scores based on local normative data were ACE-R: ≥82 (for ages 60–69, ages 70–75 ≥ 78), ACE-R MD: ≥19 (for ages 60–69, ages 70–75 ≥ 17) (Ospina-Garcia, 2015), RAVLT: 4, AIVD: <8, GDS: ≤4, HIS: ≤4 (Henao-Arboleda et al., 2010; Lasprilla, 2015). Participants in the control group were selected based on their self-reported good health, which was confirmed by a clinical interview and their performance on the screening measures.

Following the classification and validation of the participants’ SES, and the determination of the presence of aMCI according to clinical criteria, a group of 52 subjects with an aMCI and a control group of 50 participants were established. The sociodemographic and clinical characteristics of these groups are outlined in Table 1.

**Table 1.** Sociodemographic and functional characteristics of the participants.

Characteristic	Control (*n* = 50)	aMCI (*n *=* *52)			
	Mean (*SD*)	Mean (*SD*)	df	*t*	*p*
Age	72.52 (7.18)	74.73 (7.97)	100	−1.468	.145
Years of education	7.20 (3.28)	6.62 (3.29)	100	1.518	.132
GDS	2.10 (1.43)	2.26 (1.61)	100	−.834	.406
Hachinski	1.22 (1.01)	0.98 (0.82)	100	.586	.559

*Note*. aMCI = amnestic mild cognitive impairment; GDS = The Geriatric Depression Scale; SD = standard deviation. The Physical Self-Maintenance Scale and the Instrumental Activities of Daily Living Scale tests have a variance equal to 0, so they are not included in the table.

A priori power analysis was conducted using G*Power version 3.1.9.7 (Faul et al., 2007) to ensure an adequate sample size. The results indicated that, for a medium effect size (Cohen’s *d* = 0.50), an alpha error probability of *α* = 0.05, and a two-group independent samples design, a total sample size of 102 participants would be sufficient to achieve a power of 80%.

### Instruments

A semi-structured interview was conducted to assess the general health and relevant clinical history of the participants. The assessment protocol comprised cognitive assessment questionnaires, including the ACE-R (Mioshi et al., 2006), the RAVLT (Bean, 2011; Rey, 1958), the Rey–Osterrieth Complex Figure Test (ROCF) (Berry et al., 1991), the phonological and semantic verbal fluency tasks (Nass, 1984), the Trail Making Test (TMT) Form A and B (Bucks, 2013), and an abbreviated version of the Boston Naming Test (Roth, 2011) consisting of 20 items. In addition, as previously stated, the following functional questionnaires were utilized: IADL (Lawton & Brody, 1969), PSMS (Pfeffer et al., 1982), HIS (Pantoni & Inzitari, 1993), and GDS (Yesavage et al., 1982).

In order to estimate the level of CR, the Cognitive Reserve Index questionnaire (CRIq) was employed, as developed by Nucci et al. (2012). This instrument, consisting of 20 items, assesses three areas through questions about life habits and experiences: (1) Education, encompassing school attendance and participation in additional courses; (2) work activity, categorized by job held, level of responsibility, and intellectual demand; and (3) leisure time, including attendance at cultural events, such as theatres, museums, and cinemas, and reading. Each of these domains is assigned a score, and a total score is subsequently calculated to reflect the individual’s overall CR level. According to Nucci et al. (2012), the final CRIq result is divided into five levels: low (less than 70 points), low-medium (70–84 points), medium (85–114 points), medium-high (115–130 points), and high (more than 130 points). A trained psychologist administered all tests, including the CRIq. In clinical cases of aMCI, a reliable informant familiar with the participant’s background was consulted to ensure accurate data collection where necessary.

### Statistical analysis

An independent samples *t*-test was conducted to examine differences in mean scores between the two groups on the study variables. The effect size was calculated under the assumption of equal variances, and the significance level was set at 0.05 for all analyses. The central results of the *t*-test (*t*, *df*, and *p*-value) and the associated effect size are reported when differences were significant (Cohen’s *d* = 0.29 small, 0.49 medium, 0.69 large). Pearson correlation was performed within each group to analyze the relationships between CRIq (CRI-Total, CRI-Education [CRI-Ed], CRI-WorkingActivity [CRI-WA], CRI-LeisureTime [CRI-LT]) and overall cognitive performance as measured by the applied tests. A binomial logistic regression was conducted using CRI-Total as a predictor to assess its moderating effect on aMCI in the study subjects. The procedures were performed using SPSS v25. Finally, a receiver operating characteristic (ROC) curve was generated to estimate the discriminative capacity of CR between cases and controls.

## Results

Table 1 provides a summary of the sociodemographic and functional characteristics of the participants. No statistically significant differences were observed between the groups with respect to age, years of education, or gender distribution (*χ*^2^(1, *n* = 102) = 0.594, *p* = .44). Additionally, the absence of clinical depression and signs of vascular dementia was observed in both groups, with no statistically significant differences between the groups in these domains. All participants achieved adequate scores on functional activities scales (IADL-PSMS), both in the control group and in the aMCI group (mean = 8.00, SD = 0.00). In addition, all participants were classified as having SES, specifically at levels E (49 subjects) and D (45 subjects) on the ESOMAR scale.

The results of the cognitive tasks demonstrated that individuals diagnosed with mild cognitive impairment (aMCI) exhibited significantly poorer performance in comparison to the control group across most cognitive domains (see Table 2). Specifically, significant differences were found in global cognition as measured by the ACE-R (*t* = 13.78, *p* < .001), memory (ACE-R DM *t* = 13.10, *p* < .001), verbal memory (TAVR *t* = 10.55, *p* < .001; TAVR-DR *t* = 4.22, *p* < .001), visuospatial memory (ROCF DR *t* = 2.18, *p* = .31), attention (TMT-A *t* = −3.55, *p* < .001; TMT-B *t* = −2.74, *p* < .001), and verbal fluency (*t* = 1.97, *p* = .051). No statistically significant differences were observed in the general visuospatial domain, naming, and semantic fluency.

**Table 2.** Comparison of means for participants’ cognitive performance and cognitive reserve.

Cognitive domains	Control (*n *=* *50)	aMCI (*n *=* *52)				
	Mean (*SD*)	Mean (*SD*)	df	*t*	*p*	Cohen’s *d*
ACE-R	89.76 (3.67)	80.62 (2.71)	100	13.78	<.001	2.73
ACE-R MD	22.98 (1.81)	18.09 (1.18)	100	13.10	<.001	2.60
RAVLT	6.44 (1.26)	4.11 (0.94)	100	10.55	<.001	2.09
RAVLT-DR	5.30 (1.85)	3.69 (1.98)	100	4.22	<.001	.836
ROCF	23.24 (6.92)	21.75 (5.87)	100	1.31	.191	
ROCF-DR	11.94 (5.30)	9.80 (4.55)	100	2.18	.031	.432
TMT-A	85.40 (35.27)	111.90 (40.62)	100	−3.55	<.001	−.695
TMT-B	167.08 (80.60)	220.48 (72.66)	100	−3.51	<.001	−.697
Boston Naming Test	17.38 (2.15)	16.65 (2.17)	100	1.69	.094	
VF	13.28 (2.49)	12.30 (2.47)	100	1.97	.051	.391
SF	15.52 (2.54)	14.59 (2.66)	100	1.79	.076	
CRI-Education	96.48 (17.14)	85.44 (14.62)	100	3.502	<.001	.694
CRI-WorkingAcitivity	100.50 (18.36)	88.80 (16.50)	100	3.385	.001	.670
CRI-LeisureTime	95.54 (25.64)	80.92 (24.54)	100	2.740	.007	.543
CRI-Total	96.22 (18.97)	80.53 (18.15)	100	4.265	<.001	.845

*Note*. ACE-R = Addenbrooke Cognitive Examination Revised (MD = Memory Domain); CRI = Cognitive Reserve Index; *p* = significance level; RAVLT = Rey Auditory Verbal Learning Test (DR = Delayed Recall); ROCF = Rey–Osterrieth Complex Figure Test; *SD* = standard deviation; SF = semantic fluency; *t* = Student’s *t*; TMT = Trail Making Test; VF = verbal fluency.

The results of the CRI indicate that the control group obtained significantly higher scores across all dimensions when compared to the aMCI group. Specifically, statistically significant differences were identified in CRI-Ed (*t* = 3.130; *p* = .002), CRI-WA (*t* = 3.123; *p* = .002), CRI-LT (*t* = 2.273; *p* = .025), and the total CRI score (*t* = 3.726; *p* < .001).

Table 3 presents the distribution of participants based on their clinical condition and the classification of total CRIq scores as defined by Nucci et al. (2012). A chi-square test indicated that there were significant differences in the distribution of total CRI scores between the groups (*χ*^2^=13.99, *p* = .007).

**Table 3.** Description of participants’ total cognitive reserve index levels according to the classification by Nucci et al. (2012).

Variable	High	Medium–high	Medium	Medium–low	Low
Control (*n *=* *50)	5%	14%	50%	19%	12%
aMCI (*n *=* *52)	2%	0%	38%	25%	35%

*Note*. aMCI = amnestic mild cognitive impairment.

**Table 4.** Correlation between cognitive performance and cognitive reserve index across groups.

Cognitive domain	Control (*n* = 50)				aMCI (*n* = 52)			
	CRI-Ed	CRI-WA	CRI-LT	CRI-Total	CRI-Ed	CRI-WA	CRI-LT	CRI-Total
ACE-R	.045	−.012	−.005	−.009	.294*	.170	.301*	.367**
ACE-R MD	.255	.002	.019	.114	−.021	−.038	−.058	−.049
RAVLT	.192	−.098	−.009	.054	−.062	.020	.031	.015
RAVLT-DR	.029	.153	.098	.127	.234	.102	.328*	.327*
ROCF	.024	−.041	.127	.082	.148	.337*	.099	.265
ROCF-DR	.081	−.127	.018	.032	.273	.383**	−.063	.245
TMT-A	−.025	−.050	−.188	−.142	.040	−.221	−.118	−.153
TMT-B	−.038	−.099	−.169	−.127	.128	−.267	−.016	−.086
Boston Naming Test	.262	.277	−.102	.154	−.026	.021	.342*	.215
VF	.206	.051	.034	.111	.082	−.099	.132	.083
SF	.236	.038	−.024	.088	.136	.045	.342*	.286*

*Note*. ACE-R = Addenbrooke Cognitive Examination Revised (MD = Memory Domain); aMCI = amnestic mild cognitive impairment; CRI = Cognitive Reserve Index; CRI-Ed = CRI-Education; CRI-LT = CRI-LeisureTime; CRI-WA = CRI-WorkingAcitivity; RAVLT = Rey Auditory Verbal Learning Test (DR = Delayed Recall); ROCF = Rey–Osterrieth Complex Figure Test; SF = semantic fluency; TMT = Trail Making Test; VF = verbal fluency.

* *p* < .05.

** *p* < .01.

The correlations between the CRIq indices and general cognitive performance for both the control group and the group with aMCI are presented in Table 4. No significant correlations were identified in any category within the control group. Conversely, within the sample of older adults with aMCI, CRI-Ed, CRI-WA, and CRI-LT exhibited positive correlations with scores from the ACE-R, TAVR-DR, Boston-NT, and SF scores.

### Binomial logistic regression

A binomial logistic regression was conducted with CRI-total as the independent variable and aMCI as the dependent variable. Two regression models were computed: one using the total CRI score and another with the three individual measures separately. The model utilizing the total score yielded a significant result (Δ*Χ*^2^ = 16.954; *p* < .001), accounting for approximately 20% of the variance (Nagelkerke *R*^2^ = 0.204). In this case, the total CRI score was found to be a significant predictor of aMCI (OR = 0.955; WS = 13.583; *p* < .001).

The second model was calculated using independent scores. This model was found to be generally significant with the three variables (Δ*Χ*^2^ = 20.193; *p* < .001) and explains approximately 25% of the variance (Nagelkerke *R*^2^ = 0.240). However, it should be noted that only the CRI scores related to work (OR = 0.970; WS = 4.939; *p* < .001) and school (OR = 0.546; WS = 14.410; *p* < .001) were found to be significant.

**Figure 1.**
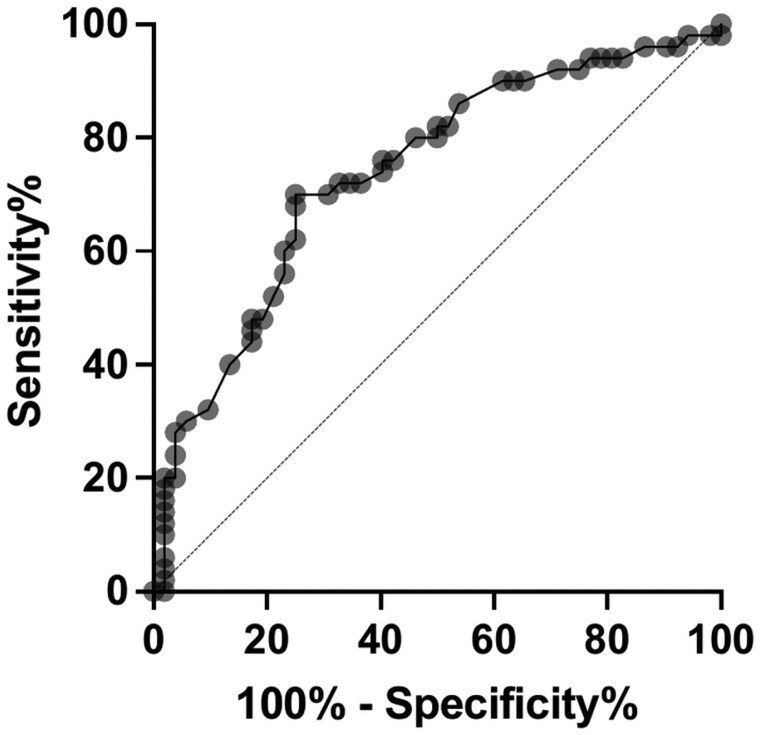
Receiver operating characteristic curve of cognitive reserve index-total and amnestic mild cognitive impairment.

Finally, the results of the ROC curve (see Figure 1) indicate that the model has adequate discriminatory capacity, with an area under the curve (AUC) of 0.738 (95% CI: 0.642 to 0.835; *p* < .0001), reflecting a significant difference between the case and control groups. The optimal cutoff point, determined using Youden’s index, was found to be 90.50 points on the CRI Total scale, with a sensitivity of 70% and a specificity of 75%.

## Discussion

In this study, we sought to identify and analyze differences in cognitive reserve in a sample of low SES older adults with and without aMCI. The hypothesis was tested, and the results indicated that CR may have an impact on cognition in subjects with low SES. The findings of this study offer the opportunity to conclude that: (1) low CR is indicative of pathological cognition in subjects with low SES. (2) A higher level of CR in subjects with low SES, even if not meeting the criteria for High CR as defined by Nuci et al. (2012), has a role in mitigating aMCI in our sample.

In recent decades, there has been a significant amount of research focusing on the role of CR in healthy older adults with different types of aMCI and AD. While various studies have proposed that variables such as education, occupation, lifestyle habits, and SES are involved in the concept of CR, a review of the literature reveals a paucity of studies that have examined the distribution of CR in healthy and low SES aMCI populations. The works of Sohrabi et al. (2023) and Yasuno et al. (2020) included an estimation of SES, but in neither case specifically aimed to characterize the distribution or influence of low SES on CR in this type of population. Along these lines, Meyer et al. (2018) conducted a longitudinal study to explore the impact of neighborhood socioeconomic status (NSES) on cognitive function. Their results indicated that NSES was associated with better performance on cognitive tests. However, their study did not include an objective measure of CR and took an environmental approach to studying SES, in contrast to our research, which takes an individual perspective. Nevertheless, it is important to recognize that when examining the impact of SES on cognition and CR, consideration must be given to the interaction between environmental and individual variables, particularly when studying populations with limited access to resources.

In the present study, the proportion of healthy or aMCI subjects exhibiting high or medium-high CR levels is negligible, and slightly higher in the control group. In general, the healthy subjects are concentrated around a medium level of CR. By contrast, a higher percentage of subjects with aMCI fell into a low or medium/low level of CR. A similar result was observed in the overall average CRI-total result. The mean and distribution of CR reported in this study differ from those reported in other studies with international populations that have estimated CR using the CRIq, where the mean and percentage of participants with high or medium-high levels of CR tend to rank high or medium-high (Marselli et al., 2024; Martinčević & Vranić, 2023; Porricelli et al., 2024). However, as these studies do not differentiate by SES among their participants, comparisons with our results are limited. Furthermore, a potential second bias may be introduced when considering their comparison with our distribution according to the sociodemographic and cultural differences between the population types.

The findings of this study suggest that, although the magnitude of the discrepancy observed in CR between healthy subjects and those with aMCI is modest, this disparity appears to function as a compensatory mechanism against cognitive impairment in this sample. These findings are consistent with studies indicating a positive association between CR and cognitive performance in participants (Marselli et al., 2024; Soldan et al., 2017; Xu et al., 2020). This finding suggests that, while a higher cognitive reserve does not prevent the progression of neurodegenerative pathology, it may reduce the clinical manifestation of its effects.

This assertion is substantiated by the results of the correlation analysis, which revealed a positive and significant association between CR and global cognitive functioning, as well as other cognitive tasks, exclusively within the cohort of patients diagnosed with aMCI. It has recently been interpreted (Marselli et al., 2024) that this pattern of correlation between global cognition and CR is an indicator of the process of *neural compensation* in people with aMCI. This process is theorized to involve the activation of additional neural networks or cognitive strategies that are not typically demanded by the task at hand. This additional activation of brain and cognitive resources, in contrast to the performance of healthy subjects, is not necessarily indicative of successful performance.

The application of regression analysis to the available data indicated a significant and substantial association between CRIq-Total and aMCI in our sample. This finding is consistent with the hypothesis that higher cognitive reserve is associated with a lower prevalence of aMCI. While CRI-Total does not fully explain the variability in aMCI, it is a significant predictor. The second regression model, which utilized the components of CRI, demonstrated that CRI-WA and CRI-Ed exerted the most substantial influence on the probability of averting or postponing aMCI. Of particular note is the observation that CRI-Ed exerts the most substantial influence among the measured CR factors. This finding underscores the pivotal role of higher educational attainment and engagement in relevant activities in mitigating the impact of cognitive decline later in life. This finding aligns closely with the conclusions of previous studies (Avila et al., 2021; Mungas et al., 2018; Staekenborg et al., 2020), which have reported education as a significant predictor of CR.

The result of the ROC curve analysis indicates an adequate level of discrimination and a satisfactory balance between the sensitivity and specificity of the model in discriminating between healthy subjects and subjects with aMCI based on CR level. This suggests that CR can be a useful measure in determining the risk of aMCI. The optimal cutoff point corresponds to a medium level of CR on the CRI-q, which confirms our previous finding that in low SES subjects, a medium level of CR is sufficient to protect against cognitive impairment in subjects with low SES.

The present study is subject to certain limitations, which are related to the relatively small sample size. This is partly due to the difficulty of recruiting volunteers from this type of population. Secondly, to expand our results, it is necessary to compare different samples from various socioeconomic levels, particularly with a sample equivalent to a high SES. This will enable us to develop a more comprehensive comparative framework to help us determine the potential influence of SES and sociodemographic factors on CR in healthy and pathological ageing.

It is important to note that the present study focused on analyzing SES at the current stage of the participants’ life cycle. Recent literature on this topic (Greenfield et al., 2022; Zhang et al., 2020), has shown that low SES during childhood and adolescence can influence cognitive health later in life. Therefore, the cognitive patterns observed in older adults with low SES, as described in this study, are likely to result not only from the development of cognitive reserve (CR) through adult habits, but also from structural conditions throughout the life cycle in which social, environmental, and individual variables are closely intertwined.

This directly raises a key challenge in current CR research: how to operationalize this construct while accounting for the complex interactions among variables, particularly those linked to SES throughout the life course. As highlighted by Harrison et al. (2015), considerable variability remains in the definition and measurement of CR. In our study, we opted for a multidimensional tool —the CRIq— that has been widely applied and standardized across 14 cultures (Kartschmit et al., 2019), alongside a rigorous SES classification. However, we acknowledge that there is still a gap remaining in CR theory and its operationalization as a life-course construct, particularly regarding the potential influence of early-life conditions and other socio-environmental factors on cognitive outcomes later in life. Closing this gap is crucial for achieving a more comprehensive understanding of CR across diverse populations.

In conclusion, we have used a standardized SES measure to document the distribution of CR in a sample of healthy older adults with low SES and aMCI. Despite low levels of education and overall CR, the findings suggest that CR still has a protective effect on this demographic group. Future studies should explore the relationship between CR and SES within the context of brain-behavior associations by integrating neuroimaging techniques. This will help to clarify whether structural or functional differences are associated with CR in low SES populations, providing direct insight into the compensation and resilience processes of the associated neural networks. In addition, sample sizes should be increased to enable ­comparisons between high and low SES populations and to facilitate the exploration of AD biomarkers, such as the A/T/N framework.

## Data Availability

The data that support the findings of this study are available from the corresponding author, J. F. Martínez Flórez, upon reasonable request.
